# Asthma Across Age: Insights From Primary Care

**DOI:** 10.3389/fped.2019.00162

**Published:** 2019-05-03

**Authors:** Alan Kaplan, Antony Hardjojo, Shaylynn Yu, David Price

**Affiliations:** ^1^Department of Family and Community Medicine, University of Toronto, Toronto, ON, Canada; ^2^Observational and Pragmatic Research Institute, Singapore, Singapore; ^3^Division of Applied Health Sciences, Centre of Academic Primary Care, University of Aberdeen, Aberdeen, United Kingdom; ^4^Optimum Patient Care, Cambridge, United Kingdom

**Keywords:** asthma, guidelines, primary care, children, adult, phenotypes, diagnosis, management

## Abstract

Asthma is a heterogeneous disease comprising of multiple phenotypes and affects patients from childhood up to old age. In this review, we summarize the current knowledge on the similarities and differences in asthma across different age-groups, with emphasis on the perspective from primary care. Despite the similar disease presentation, phenotyping studies showed that there are differences in the distribution of phenotypes of asthma presenting in childhood compared to that in adulthood. Whereas, asthma with early age of onset tends to be of the atopic phenotype, the disease shifts toward the non-atopic phenotypes at later ages. Studies within primary care patients aiming to elucidate risk factors for future asthma exacerbation have shown pediatric and elderly patients to be at higher risk for future asthma attacks compared to other adult patients. Regardless, both pediatric and adult studies demonstrated previous asthma episodes and severity, along with high blood eosinophil to predict subsequent asthma attacks. Differences in childhood and adult asthma are not limited to the underlying phenotypes but also extends to the challenges in the diagnosis, treatment, and management of the disease. Diagnosis of asthma is complicated by age-specific differential diagnoses such as infectious wheezing and nasal obstruction in children, and aging-related problems such as heart disease and obesity in the elderly. There are also age-related issues leading to decreased disease control such as non-adherence, tobacco use, difficulty in using inhalers and corticosteroid-related side effects which hinder asthma control at different patient age-groups. Several clinical guidelines are available to guide the diagnosis and drug prescription of asthma in pediatric patients. However, there are conflicting recommendations for the diagnostic tools and treatment for pediatric patients, posing additional challenges for primary care physicians in working with multiple guidelines. While tools such as spirometry and peak flow variability are often available in primary care, their usage in preschool patients is not consistently recommended. FeNO measurement may be a valuable non-invasive tool which can be adopted by primary physicians to assist asthma diagnosis in preschool-age patients.

## Introduction

The term “asthma” encompasses1 heterogeneous phenotypes of conditions sharing similar symptoms yet different underlying causes and prognosis (1, 2). Depending on the age of presentation, symptoms of asthma may represent different phenotypes of the disease, each with its own challenges in the diagnosis, management, and treatment. A better understanding of the different subsets of asthma is hoped to assist us to better diagnose, manage, and treat this disorder.

Primary care represents the frontline of patient management and not all patients with asthma require referral to secondary care (3). Thus, this article aims to describe the age-related phenotypes of asthma and the challenges presented by asthma across different age groups, with emphasis on insight from primary care.

## Asthma Across Ages

### Phenotypes of Asthma

The traditional method of asthma phenotyping involves grouping of asthma by characteristics such as the age of onset, trigger, atopic status, and presence of biomarkers, in a so-called “biased” approach to phenotyping (4–6). It is well-known that early-onset asthma represents a distinct phenotype compared to adult-onset asthma. Adult-onset asthma may also be further divided into long-standing asthma, asthma which remitted at childhood and subsequently relapsed, and new onset adult asthma (7).

Cluster analysis presents an unbiased statistical approach to phenotype asthma by grouping them into clusters with maximum similar characteristics within clusters and minimum similarity between clusters (Table 1). Haldar et al. conducted a cluster analysis from both patents with milder asthma presenting in primary care, as well as patients with refractory asthma diagnosed in secondary care (2). The study identified 3 and 4 separate clusters of asthma from the respective populations, 2 of which were identified from both populations. Two clusters denoted with early onset were both accompanied with positive atopic status, one of which had minimal signs of eosinophilic inflammation. Clusters of late-onset asthma include a cluster of obesity-related non-eosinophilic asthma predominant in females and a male-predominated cluster predominantly marked by active eosinophilic inflammation but fewer symptoms. Both late-onset clusters also consisted of lower proportion of patients with positive atopic status compared to the early onset clusters.

**Table 1:** Clusters of asthma phenotypes.

**Population analyzed**	**Additional patient details**	**Clusters Identified**
**Haldar et al. (2)**		
1. Primary care (*n* = 184)	Age range: 18–65 years	1. Early-onset atopic (*n* = 61) 2. Obese, non-eosinophilic (*n* = 27) 3. Benign (*n* = 96)
2. Secondary care (*n* = 187)	Age range: not specified	1. Early-onset atopic (*n* = 74) 2. Obese, non-eosinophilic (*n* = 23) 3. Early symptom predominant (*n* = 22) 4. Inflammation predominant (*n* = 68)
**Moore et al. SARP study, 2010 (8)**		
12–80 years old (*n* = 726), 304 fulfilled criteria for severe asthma	Primary/secondary: not specified	1. Early-onset atopic, normal lung function and low healthcare utilization (*n* = 110) 2. Early-onset atopic, higher medication requirement (*n* = 321) 3. Late-onset, less-atopic, obesity-related, lower lung function, and more daily symptoms and healthcare utilization (*n* = 59) 4. More severe, long-standing, early-onset, atopic, reversible lung obstruction, high ICS usage (*n* = 120) 5. More severe, long-standing, late-onset, less-atopic, less-reversible lung obstruction, high ICS usage (*n* = 116)
**Schatz et al. TENOR study, 2014 (9)**		
6–11 years old (*n* = 518)	Patients recruited from “managed care organizations, community physicians or group practices, and academic centers”	1. White race with no tobacco exposure (*n* = 115) 2. Female cluster (*n* = 81) 3. Non-atopic (*n* = 162) 4. Passive smoke exposed (*n* = 87) 5. Non-white race (*n* = 73)
≥12 years old (*n* = 3,612)		1. White female adult onset, low IgE (*n* = 1262) 2. High atopic cluster (*n* = 659) 3. Male cluster (*n* = 664) 4. Non-white cluster (*n* = 596) 5. Non-white race (*n* = 431)

*ICS, Inhaled corticosteroid*.

A cluster analysis which combined severe asthma and non-severe asthma patients recruited into the Severe Asthma Research Program (SARP) identified 5 clusters of asthma (8). Similar to the characteristics of the clusters identified by Haldar et al., clusters associated with early age of onset were also associated with higher atopic status while those with later-onset were less atopic. Another cluster analysis study was conducted on children (6–11 years) and adolescent to adult patients (>11 years) with severe or difficult-to-treat asthma from the TENOR (The Epidemiology and Natural History of Asthma: Outcomes and Treatment Regimens) study (9). Five clusters were identified within both age-groups differentiated by demographics, a topic status, tobacco exposure (in children), and aspirin sensitivity (in adolescent to adults). Interestingly, in contrast to adolescent and adult patients, none of the five children clusters had significantly different asthma outcomes (within the next 12 months) among each other, though this is likely due to the population of the study being limited to severe asthma patients.

**Figure 1:**
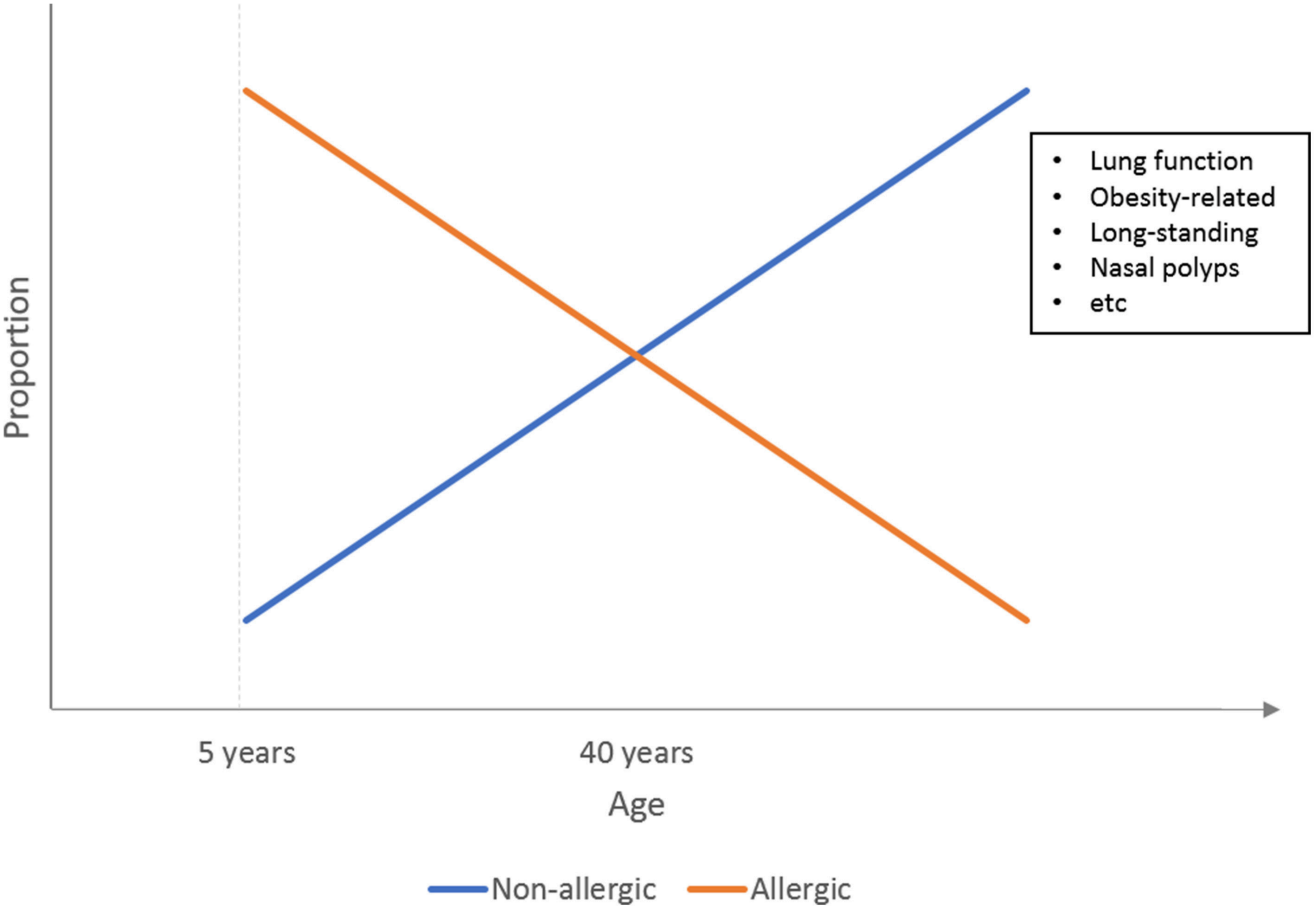
Illustration of the relationship between asthma phenotypes and the age of onset. At younger age, asthma is predominated by the atopic phenotypes, which are gradually phased out by non-atopic phenotypes such as obesity-related asthma at adulthood.

Results from both classic “biased” phenotyping studies and cluster analysis studies thus confirmed that early onset asthma is more likely to be atopic in nature, while adult-onset asthma tends to have non-atopic causes, primarily obesity. Figure 1 illustrates a simple representation of this relationship between the atopic status of asthma and age as previously reviewed by Wenzel et al. (6).

Current asthma guidelines state that more research on the value of asthma phenotyping to guide treatment is required (10, 11).

### Severe Asthma Across the Age

An estimated 5–10% of asthma patients suffer from a severe form of asthma (12). Despite the rarity, severe asthma poses high healthcare resource burden (13). The GINA (Global INitiatives for Asthma) guideline defined severe asthma as asthma which requires high dose ICS (inhaled corticosteroid)/LABA to prevent it from being uncontrolled or asthma which remains uncontrolled despite this treatment (10). Regardless, there is currently no universally accepted definition for severe asthma, preventing accurate estimation of its global prevalence, which is further contributed by the absence of a global registry for this subset of asthma until the recent creation of the International Severe Asthma Registry (14).

A recent review by Guilbert et al. highlighted the differences between adult and pediatric severe asthma (15). Compared to the adult counterpart, severe asthma in children had a more rapidly changing phenotype. They were also characterized with higher exhaled nitric oxide, IgE, and eosinophil levels. Despite the differences in pediatric and adult severe asthma, the current treatment guidelines for pediatric severe asthma is currently based on extrapolation from adult studies (15). Thus, there is a need for better guidelines on severe childhood asthma.

## Risk Prediction of Asthma

Asthma exacerbation is a major cause of quality of life disruption and healthcare resource consumption. Thus, a part of asthma management includes managing the risk for future asthma exacerbations/attacks on top of maintaining symptom control (10, 16). The GINA provides a guideline for assessment of asthma exacerbations in primary care setting (10). The guideline suggests documentation of symptom histories such as the onset, severity, potential risk factors, and current therapy, along with physical examination and objective measurements such as pulse oximetry and peak expiratory flow measurement (for patients > 5 year).

Numerous studies have been conducted to investigate potential risk factors for future asthma exacerbations (Table 2). Observational studies using primary care data have identified factors such as previous asthma attacks and medication usage in and biomarkers such as blood eosinophil to predict exacerbations in subsequent years (17, 18).

**Table 2:** Risk prediction of future asthma exacerbations/hospitalization^*^.

**References**	**Population**	**Age**	**Definition of asthma**	**Outcome**	**Predictive factors**
**PEDIATRIC**					
Swern et al. (20)	Patient from double-blinded multicenter RCT	2–5 years	Physician-diagnosed asthma: At least 3 episodes of asthma symptoms such as cough, wheeze, shortness of breath	Asthma attacks	• Daytime cough • Daytime wheeze • Night-time β2-agonist
Bloom et al. (3)	Primary care	<5 years and 5–17 years	Patients with read codes for asthma	Annual asthma exacerbation rate and time to first exacerbation	• Higher asthma severity
Haselkorn et al. (21)	Severe asthma children recruited from “Managed care organizations, community physicians or group practices, and academic centers”	6–11 years	Diagnosed with severe asthma or mild/moderate asthma considered to be difficult-to-treat by site specialist.	Future severe exacerbation	• Recent exacerbation • Non-white race (vs. white) • 3-4 allergic triggers • Poor asthma control
Covar et al. (22)	Patient from double-blinded multicenter RCT	6–14 years	Mild-moderate persistent asthma: Diary-reported symptoms or β-agonist use (not including pre-exercise), or mean morning and evening peak flow <80%.	Asthma exacerbation	• Baseline exacerbation
Turner et al. (19)	Primary care	5–12 years	Read Code for asthma diagnosis.	Asthma attack in 1 year follow-up	• Higher GINA management step • Consultation for LRTI • Blood eosinophil >400/μL • Baseline asthma attacks • Younger age • Lower peak expiratory flow
Engelkes et al. (24)	Primary care	5–18 years	Algorithm-validated from list of patients with ICD code and free-text of asthma	Severe asthma exacerbation	• Younger age • Exacerbations in the previous year • Use of any asthma medication
				Time until next exacerbation	• Younger age • Female gender • Exacerbations in the previous year • Respiratory infection in the previous year • Asthma specialist visit in the previous year • ICS prescription • Blood eosinophil >300/μL • Comorbid eczema
**ADULTS**					
Bloom et al. (3)	Primary care	18–54 years and ≥55 years	Patients with Read Codes for asthma	Annual asthma exacerbation rate and time to first exacerbation	• Higher asthma severity
Kerkof et al. (25)	Primary care patients with secondary care data linkage	≥5 years	Active asthma defined as having diagnostic Read Codes for asthma, no code for resolved asthma, and at least 2 prescriptions for asthma.	Asthma-related hospital readmission 1 year after discharge	• Blood eosinophil count ≥0.35 × 10^9^ cells/L
Blakey et al. (17)	Primary care	12–80 years	Active asthma defined as having diagnostic Read Codes for asthma, no code for resolved asthma, and at least 2 prescriptions for asthma.	Asthma exacerbations in 2 years follow-up period	• Baseline (1 year) asthma exacerbations • Older age, female gender, current smoking, overweight • Co-morbid rhinitis, eczema, GERD, nasal polyps, or anaphylaxis • High blood eosinophil count • Higher daily SABA dose • NSAID, LTRA or LABA prescriptions • More acute OCS course • More asthma-related hospitalization • More primary care visits • Lower PEF%
Price et al. (18)	Primary care	12–80 years	Patients with recorded physician-diagnosis for asthma and no other chronic respiratory diseases.	≥2 severe asthma exacerbations in 1 year follow-up	• Older age, female gender, current smoker, overweight, • Blood eosinophil >400/μL • Co-morbid anxiety, diabetes, eczema, GERD, rhinitis • NSAID prescriptions • Higher asthma treatment step (BTS-SIGN) • General practice consultation for LRTI • Outpatient asthma attendance • Having acute OCS courses • >400 μg/day SABA dose • >800 μg/day ICS dose (FP equivalent)

* *Studies which analyzed pediatric and adult patients as a single group are categorized as adult studies. BTS, British Thoracic Society; FP, Fluticasone propionate; GERD, Gastroesophageal reflux disease; GINA, Global Initiatives for Asthma; ICS, Inhaled corticosteroid; LABA, long-acting β-agonists; LRTI, Lower respiratory tract infection; LTRA, Leukotriene receptor antagonists; NSAID, Nonsteroidal anti-inflammatory drugs; OCS, oral corticosteroid; PEF, Peak expiratory flow; SABA, short-acting β-agonist; SIGN, Scottish Intercollegiate Guidelines Network*.

Knowledge of the risk factors for asthma attacks in children is scarce, and even less is known in preschool asthma, where exacerbation may have viral triggers (19). Swern et al. conducted a *post-hoc* analysis of 2–5years old patients (*n* = 689) previously enrolled in a randomized control trial (20) The trial included patients with physician-diagnosed asthma defined as ≥3 episodes of asthma symptoms in the past year including but not limited to cough, wheezing and shortness of breath. The study identified a combination of daytime cough, daytime wheeze, and night-time β2-agonist use to be predictive of exacerbation in the following day. However, this study did not report risks for exacerbations further than 3 days after the identification of risk factors.

A very recent study by Bloom et al. reported the exacerbation risks on the general asthma population across different age-groups including patients under 5 years old within the UK's national electronic healthcare records (3). Interestingly, patients ≥55 years and <5 years had the highest rate for exacerbations, in comparison to adolescents (5–17 year) and adults (18–54 years). Regardless of the age groups, higher asthma severity (as defined by the BTS [British Thoracic Society] treatment steps) was a significant predictor for annual exacerbation rate and time to next exacerbation, especially within the <5 years group.

Results on older children and adolescents confirmed the findings of the adult studies, i.e., recent asthma attacks and blood/systemic eosinophilia were consistently reported as predictors for future attacks (17, 18, 21–25). A recent observational study combining data from 2 primary care databases in the UK also reported recent asthma attacks, previous consultation for lower respiratory infections, blood eosinophils > 400/μL, and younger age to be indicative of high-risk for future asthma exacerbation in children 5–12 years of age (23). Another large observational study using general practice data of Dutch children 5–18 years old reported asthma exacerbations and asthma treatment in the preceding year and younger age to be risk factors for severe asthma exacerbations (24). While blood eosinophil level of ≥300 cells/uL was not a risk factor, children with heightened blood eosinophil had shorter time till exacerbation.

**Figure 2:**
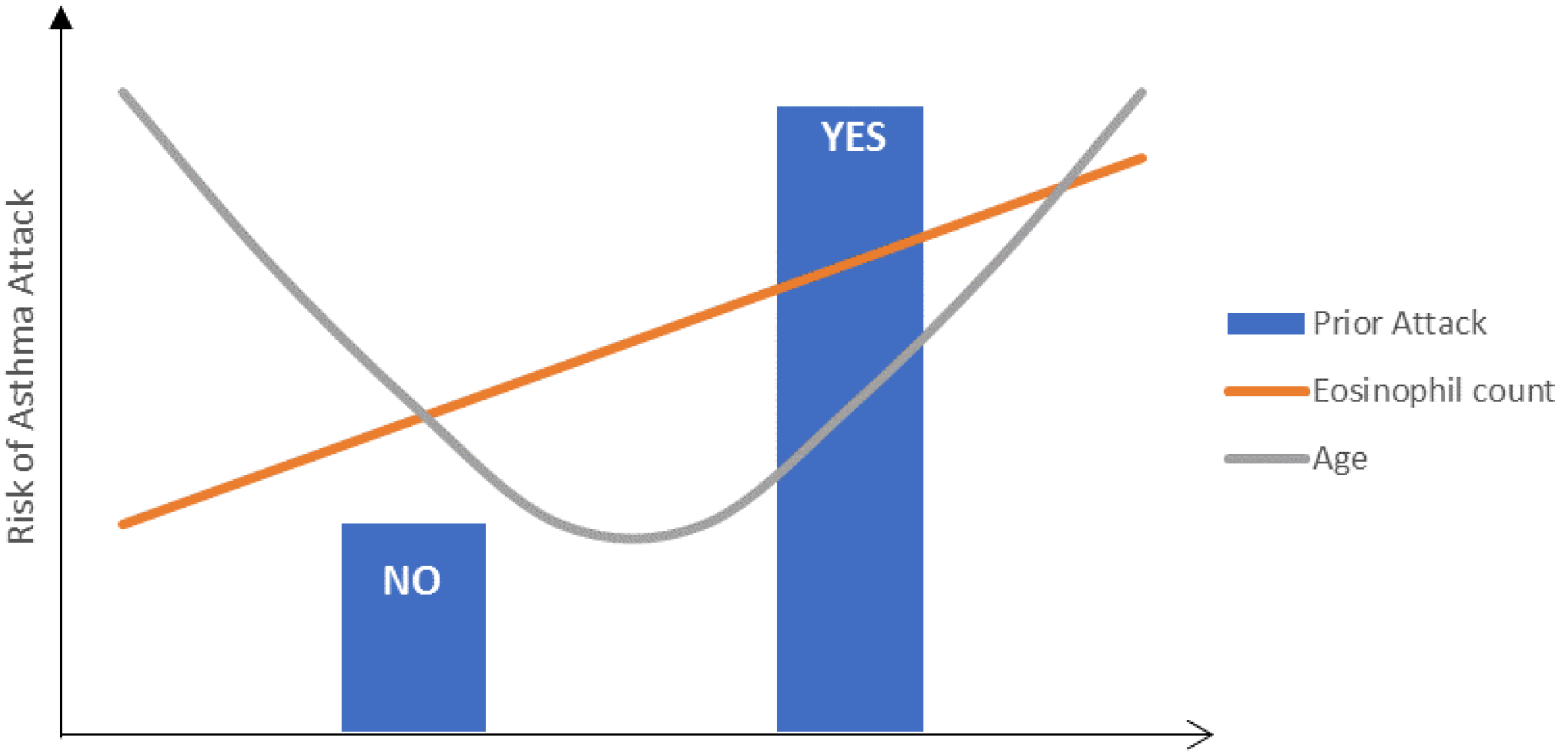
Illustration of the risk factors for future asthma exacerbations.

Interestingly, in contrast to adult population studies where older age was associated with increased risk (17, 18), studies in children reported younger age to be associated with higher risk for asthma attacks/exacerbations (23, 24). This confirms the observation by Bloom et al that patients at both ends of the age spectrum were at the highest risk for exacerbations (Figure 2) (3).

## Diagnostic Challenges Across Age

**Figure 3:**
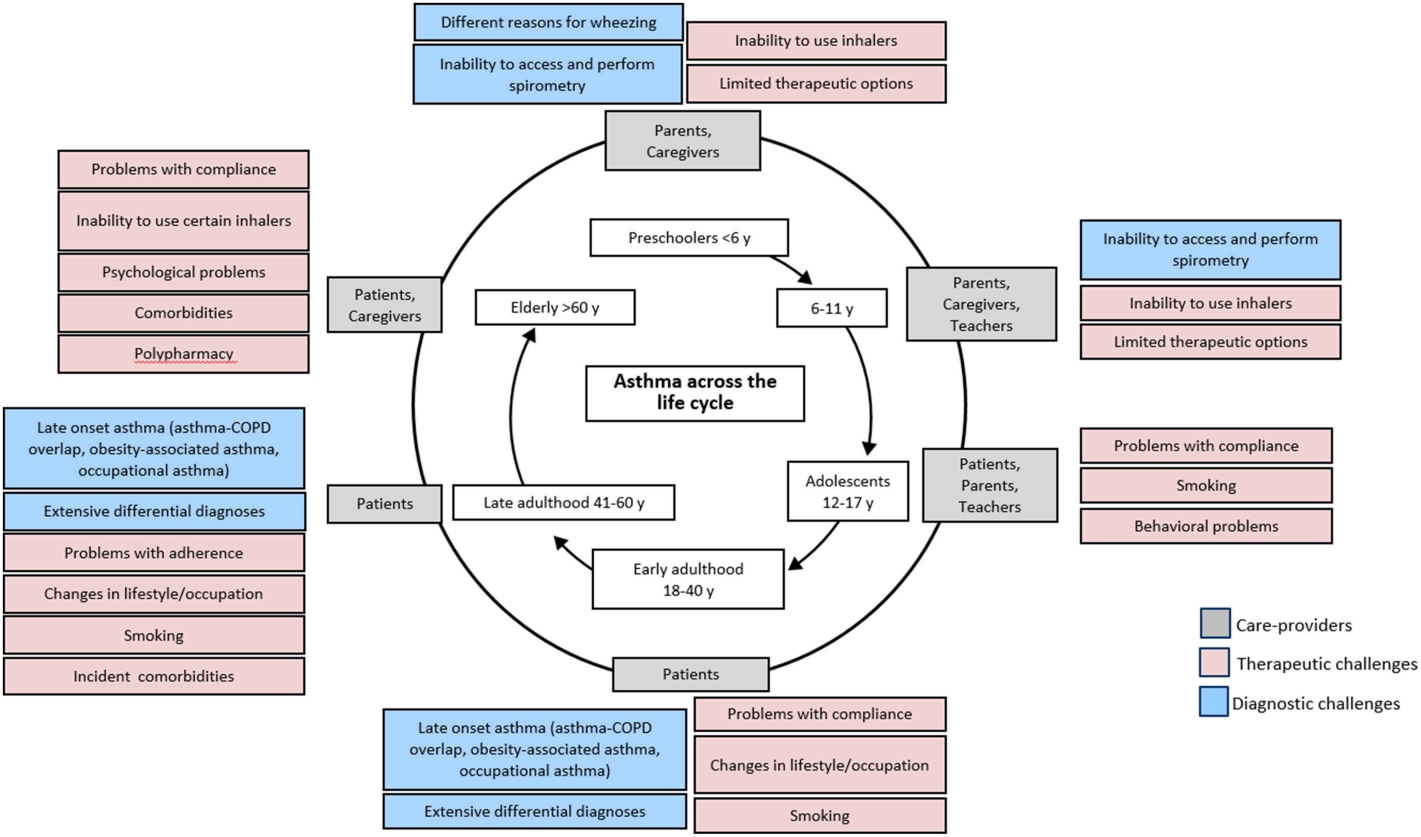
Challenges related to asthma treatment across age. Adapted with permission from: Kaplan A, Covar R, Vanderwalker M. An update on treatment options for children and adults with asthma. Manuscript in Preparation.

There is still a very clear lack of understanding of asthma in children under 5 years, and diagnosis of asthma within the preschool age is challenging due to the lack of proper guideline and definition (26). In this age group, symptoms of wheezing and cough are very common but may be the result of acute respiratory infections instead of asthma (Figure 3) (27, 28). The Tucson birth cohort (USA) identified three distinct phenotypes of wheezing in the first 6 years of life: transient early wheezing, late onset wheezing and persistent wheezing (29, 30). The result from subsequent follow-up reported that not all children who wheezed developed asthma in later childhood, although children with atopic wheezing were the most likely (30). Thus, differentiating transient symptoms from symptoms of the more persistent asthma poses a challenge in early age.

The GINA (10) and CTS (Canadian Thoracic Society) (31) guidelines recommend consideration of symptomatology, potential triggers and family history to assist in clinical decision making for this age group of patients. The guidelines additionally recommend observing for signs of airway obstruction and reversibility with inhaled bronchodilators to assist in diagnosis. Similarly, the Australian Asthma Handbook (32) and NICE guideline (33) recommend treating preschool patients with suspected asthma based on clinical observation and conduct objective tests only after 5 years of age.

Objective tools recommended by the clinical guidelines to diagnose asthma across different age groups are summarized in Table 3. Lung function tests, primarily spirometry, are recommended to confirm asthma diagnosis in school-aged children and adults within primary care practice (10, 11, 16, 33–35). However, in many countries, spirometry is not available to primary care, and conducting lung function tests on patients <5 years old is challenging due to their inability to produce consistent lung function readings (Figure 3) (10, 26, 28, 36). The BTS guideline thus does not recommend lung function measurement to guide asthma management in this age group (16). A joint statement by the American Thoracic Society (ATS) and European Respiratory Society (ERS) suggested taking only one satisfactory measurement, instead of the ideal of at least two separate measurements to accommodate pediatric patients (36). The GEMA emphasizes the need for nursing supervision and suggested measurement of FEV_0.5_ instead of FEV_1_ in preschool children 3 years and above (11).

**Table 3:** Objective tests recommendation in each age-groups and availability in primary care.

	Preschool (<5 years)^*^	**Children (5–12 years)**	**Adolescent and adults (>12 years)**	**Availability in primary care**
Peak flow variability	Not recommended by BTS	Recommended by GINA, CTS, and NICE; Not recommended by BTS	Recommended by GINA, BTS (in adults), CTS, GEMA and NICE.	Available
Spirometry	Not recommended by GINA^†^ and BTS	Recommended by GINA, BTS, CTS, GEMA, AAH, and NICE	Recommended by GINA, BTS, CTS, GEMA, AAH, and NICE	Available
FeNO	Recommended by GINA, GEMA, and BTS (for 3–4years)	Not recommended by GINA; Recommended by NICE and BTS (for eosinophilic asthma)	Not recommended by GINA; Recommended by NICE, GEMA, and BTS (for eosinophilic asthma)	Not usually available
Bronchial provocation test	Not recommended by GINA	Recommended by GINA, BTS, AAH, and CTS	Recommended by GINA, BTS, CTS, AAH, and NICE (at >17 years)	Available in speciality clinics also
Allergen sensitization	Recommended by GINA and GEMA but not conclusive (doesn't exclude nonatopic asthma)	Recommended by GINA, GEMA, and BTS but not conclusive; Not considered essential by AAH. Not recommended by NICE	Recommended by GINA, GEMA, and BTS but not conclusive Not recommended by NICE	Available through speciality referral
Chest X-ray	Recommended by GINA, BTS, and CTS	Recommended by BTS	Recommended by BTS, and by GINA (in elderly).	Typically available

* NICE guideline does not recommend any objective tests to guide asthma diagnosis in children <5years

† *According to GINA, children 4–5 years may undergo spirometry with guidance. GINA, Global Initiatives for Asthma; BTS, British Thoracic Society; AAH, Australian Asthma Handbook; NICE, National Institute for Health and Care Excellence; CTS, Canadian Thoracic Society*.

FeNO (Fractional concentration of exhaled Nitric Oxide) is an inflammatory biomarker which may indicate the presence of type 2 asthma (asthma characterized by Type-2 inflammation). The NICE (National Institute for Health and Care Excellence) (33) and GEMA (Spanish Guideline on the Management of Asthma) (11) guideline recommends FeNO measurement for asthma diagnosis. The GINA, on the other hand, do not recommend the usage of FeNO to aid asthma diagnosis in adults, with the argument that FeNO may be elevated in other respiratory conditions, and is not elevated in other asthma phenotypes such as neutrophilic asthma (10). However, the guideline recommends FeNO measurement in preschool-age patients. In agreement with this, a prospective study in a hospital setting reported elevated FeNO level during preschool to be predictive of school-age asthma (37). Additionally, a randomized control trial based on 24 primary care centers and one hospital showed that high FeNO level was associated with improved Asthma Control Questionnaire 7 items (ACQ7) score following ICS treatment (38). Therefore, FeNO measurement can be a non-invasive measure conducted in primary care practices to assist asthma diagnosis in preschool children and identifying patients who may benefit from ICS treatment.

Another available tool to assist primary care practitioners to predict whether the presenting wheezing symptoms in preschool children will develop into asthma by school age is the modified version (mAPI) (39) of the Asthma Predictive Index (API) created based on data from the Tucson cohort study (30, 40). The mAPI has been previously shown in an asthma birth cohort study to have high positive predictive capability for asthma at 6, 8, and 11 years based on mAPI score at the first 3 years of life (41).

Older age presents another diagnostic challenge distinct from that in younger patients (Figure 3). Symptoms of asthma in old age may be masked by aging-associated changes in pulmonary and other physiological functions (10, 42) and the presence of multiple co-morbid conditions (43). These factors lead to underdiagnosis of asthma within the elderly. The decreased respiratory capacity in the elderly may also make it difficult to conduct lung spirometry, as such the National Institute of Aging recommended alternative techniques which do not require inspiratory efforts such as imaging and forced oscillation (42) The GINA guideline recommends physical examination, such as electrocardiogram and chest x-ray, to aid in the diagnosis of elderly asthma in addition to the routine clinical history taking (10).

Diagnosis of asthma is further complicated by differential diagnosis for symptoms which may mimic asthma. As mentioned above, wheezing and cough in children are likely to be infectious in nature. Non-infectious, non-pulmonary related causes of cough and wheeze, such as gastroesophageal reflux, airway obstruction due to foreign bodies, and congenital heart disease, should also be ruled out before the diagnosis of asthma in children (10). In old age, age-related problems such as heart disease and obesity are the major contributors to differential diagnosis. Chronic obstructive pulmonary disorder (COPD) is also a common cause of misdiagnosis in primary care due to overlapping symptoms with asthma (44). In addition, they may occur concurrently, in a term known as asthma-COPD overlap (ACO). Careful symptom history taking and post-bronchodilator spirometry to test for reversible airway obstruction are recommended to differentiate asthma from COPD and ACOS (10).

## Therapy Issues at Different Ages

### Differences in Treatment Guidelines

The GINA guideline provides a step-wise management approach for treatment and management of asthma (10). The 2018 updated guideline still recommends as-needed short-acting β-agonists (SABA) for reliever treatment of asthma attacks and ICS as the initial controller medication for asthma, with addition of long-acting β-adrenoceptor agonists (LABA, as combination therapy with ICS), leukotriene receptor antagonists (LTRA) or stepping up of dosage as required for adolescent and adults patients above the age of 12 (10). Recommendations from other guidelines are similar (11, 16, 32–34): initial reliever with SABA, initial preventer treatment of ICS, and when needed, adding LABA in combination with ICS.

Initial treatment option for children 5–12 years follows that of older patients. However, LABA as the initial add-on is not recommended by the GINA (10) for this age group, in contrast to the BTS (16) recommendation. The CTS recommends LABA if stepping up ICS dosage fails to achieve control (34). Alternatively, tiotropium, a long-acting muscarinic antagonist (LAMA), administration by mist inhaler can be prescribed as an add-on in children ≥12 years and adults. LAMA is however not indicated for children <12 years by the GINA (10) and BTS (16) guidelines, though it is indicated for children ≥6 years in the US (45).

The GINA guideline dedicates a section outlining a step-by-step treatment guideline for children 5 years and younger, however, the current guideline is based on more limited evidence (10). Similar to older patients, (SABA) should be given as initial reliever upon presentation of wheezing. When necessary, i.e., symptoms suggestive of asthma or frequent wheezing episode, a low dose of ICS is recommended as the initial controller therapy. Similarly, GEMA (11) and Australian Asthma handbook (32) recommended initial SABA preventer with addition of low dose ICS when necessary. The BTS (16) guideline recommends SABA for reliever therapy together with low-dose ICS as the preventer, while the CTS guideline for preschool patients (31) recommends daily low-dose ICS as first-line therapy or SABA if symptoms were mild or infrequent. LTRA is recommended as an alternative to ICS by the GINA (10), GEMA (11), Australian Asthma Handbook (32), and BTS (16) but is not recommended for use by the CTS (31). If symptoms remain inadequately controlled with low-dose ICS, the GINA, GEMA and CTS guidelines recommend stepping-up to medium dose ICS, but this is not recommended by the BTS (16).

There is a lack of guideline on the treatment for asthma in elderly patients. Treatment of asthma in the elderly faces additional challenge due to poorer asthma control. However, more studies are required to determine whether this is due to decreased treatment response, difficulty with inhaler technique, or poorer adherence (43).

### Challenges in Control

Adherence to inhaled corticosteroid (ICS) treatment is a key factor for reduction of exacerbation and achievement of asthma control. Yet, non-adherence toward ICS is constantly reported to be a very common occurrence, as high as 80% among asthma patients (46, 47). Various factors influencing adherence have been previously described, including educational level and confidence in the treatment (48).

One of the factors influencing adherence includes changes in attitude across ages. Younger children depend on parental intervention for medication, thus it is not surprising that parental concerns on medication to be influential toward adherence in pediatric asthma (49, 50). Improvement of adherence in pediatric patients should focus on parents and caregivers. Interestingly, pediatric asthma therapy adherence has been reported to be inversely correlated with children's age despite the supposedly increased understanding of their condition (50, 51). This could suggest the presence of teenage-related intentional non-adherence, which may be due to several factors such as teenage rebellion, or embarrassment of using prescribed inhaler therapy due to peer pressure (48).

In elderly patients, non-adherence may stem from the patients' struggle due to memory loss coupled with the complexity of the treatment regimen (42). This issue is further exacerbated by the multiple comorbidities in elderly asthma patients which may lead to an increased number of medications, also known as polypharmacy, which subsequently impacts asthma control (52).

Another factor which may negatively impact treatment success is improper inhalation technique, a problem repeatedly reported to commonly occur regardless of the ICS device type (53, 54). The extensive list of possible DPI and MDI device errors and their association with poor asthma outcomes were recently described in a study utilizing primary care records of 7 European countries and Australia (CRITIKAL study) (54). Among the errors reported to be associated with exacerbation is insufficient inspiratory effort for DPI device. This error is well-established to be a major challenge in preschool children and elderly patients (55). An MDI device (28) or soft mist inhaler (56) with properly designed valved holding chamber is more suitable for preschool children.

Smoking has been consistently reported to hinder response to ICS therapy (57, 58), and poor asthma control was associated with smoking status based on an interview of over 10,000 primary care patients aged 12 years and older (59). The BTS guideline recommends higher ICS dose in patients who are current or ex-smokers (16). Smoking remains a global health behavioral problem from teenage to adulthood, with a median reported global prevalence of 10.7% (range 1.7–35.0%) between 2012 and 2015 (60). A recent study on the impact of ICS adherence on asthma exacerbation and control within primary care reported one-third of their patients to be active smokers (47). Tobacco smoking thus represents another “wrench in the gears” in achieving asthma control.

Despite the recommendation for ICS, concerns remain regarding the associated side-effects, which have in turn been reported to negatively impact patient adherence toward ICS treatment (61). ICS is known to be associated with various local side effects such as oral thrush (candidiasis) and hoarseness (62, 63). A previous study reported 63.3% of children under 6 years of age to be affected (64). In addition to local side effects, ICS treatment may also result in growth retardation in children (65). It is therefore recommended to use the lowest ICS dosage for this age group and to monitor for reduced growth velocity (10). The Canadian Society of Allergy and Clinical Immunology (CSACI) also recommended monitoring for adrenal suppression for children and adolescents receiving high dose ICS (66). Another potential systemic side effects of ICS include osteoporosis (which leads to bone fractures), cataracts and diabetes, which pose additional concern on ICS use in older patients (65). Patients administered high dose ICS over a long period (more than 3 months) should thus be monitored for any potential side effect (10, 16).

### Issues in Therapy Response

In addition to the challenges in diagnosis, old age poses additional challenges in the treatment of asthma due to decreased response to bronchodilator therapy (3, 67, 68). Knowledge and guidelines on elderly asthma are limited, and clinical trials tend to exclude elderly patients due to the presence of co-morbidities (43). Unlike in preschool children <5 years of age, there is still no dedicated section for elderly patients within the GINA guideline (10). It is also of relevance to understand whether the different phenotypes asthma: early onset atopic and late-onset asthma, would present with different responses to bronchodilator therapy in old age.

### Issues Working With Multiple Guidelines

As discussed in the previous section, different guidelines provide different recommendations in terms of prescriptions across patient age groups. The GINA, BTS, NICE, GEMA, and CTS guidelines recommend SABA reliever and ICS preventer as initial treatment for asthma. However, there is less consensus on the subsequent add-on therapy for asthma which remains uncontrolled after the initial therapy in younger age-groups. In children (5–12 by BTS, 6–11 by GINA and CTS), LABA is recommended by the BTS and CTS guidelines as an add-on if symptom control is not achieved with ICS but is not recommended by GINA. Additionally, in the preschool age-group, there are conflicting recommendations regarding the use of LTRA as an alternative to ICS (recommended by GINA, NICE, GEMA, and BTS, but not CTS), and regarding stepping up ICS dosage to achieve control (recommended by GINA, GEMA, and CTS, but not BTS). The NICE guideline, on the other hand, recommended stepping up to a moderate dose of ICS for 8 weeks following initial SABA.

Working with multiple guidelines with different recommendations for asthma management in childhood patients may cause an additional challenge for primary care physicians as highlighted previously by the Primary Care Respiratory Society of UK (69).

### Solutions

Despite the utility provided by subjective biomarker measures, they may be unavailable in primary care practices due to the barriers in implementation (70). Data sharing across practices which allow easier physician access to patients' clinical records, including records of past subjective measures, would provide a potential solution to circumvent this challenge. This may also enable a longer observation of patients' medical history to aid in differentiating between asthma, viral wheeze, and COPD in primary care.

To improve cross-sharing of patients' past medical records, there is a need to improve electronic medical records (EMR) systems which are often claims-based and lack uniformity between systems. A potential solution will be the creation of a uniform EMR template which brings together standardized past medical records while enabling patient self-reported information to be provided to primary care practitioners prior to consultation. Creation of a uniform EMR template can be done by utilizing research-based templates such as REDCap (71).

Moving forward, incorporation of clinical decision support systems (CDSS) to EMR systems may aid physicians in making informed clinical decisions despite conflicting treatment guidelines across age-groups (72) and guide the appropriate treatment while warning against the prescription of non-indicated drugs based on the patients' profile (73). Ultimately, a global EMR for primary care, which is capable of conducting machine-learning based on previous data to provide future recommendations, may serve to guide patient management in the lack of guidelines based on strong evidence.

## Conclusion

Phenotyping studies have shown that depending on the age of onset, symptoms of asthma can represent distinct phenotypes from asthma with later onset. Together with the changing phenotypes across age are the changing challenges for diagnosis, treatment, and control of asthma.

Guidelines for asthma management in young children and the elderly are still based on weaker evidence, despite the higher hurdles in management. Differentiating asthma from other diseases with similar presenting symptoms such as viral wheeze and COPD remains a challenge. Regardless, there are resources such as FeNO measurement and the mAPI (modified Asthma Predictive Index), and spirometry which can assist in the diagnosis of asthma for different age groups within the primary care setting. Future developments in electronic medical record systems to enable cross-sharing of clinical history and implementation of clinical decision support systems (CDSS) can potentially improve patient management across different age-groups.

## Author Contributions

All authors listed have made a substantial, direct and intellectual contribution to the work, and approved it for publication.

### Conflict of Interest Statement

AK declares participation in speaker and advisory boards of Boehringer Ingelheim, AstraZeneca, Novartis, Purdue, Sanofi Genzyme, Covis and Teva; as speaker for Grifols and Merck Frosst; in the smoking cessation website design for Johnson & Johnson; and in advisory boards of GSK, Mylan, Paladin labs, and Novo Nordisk. SY and AH are employees of Observational and Pragmatic Research Institute Pte Ltd, which has conducted paid research in respiratory disease on behalf of the following organizations in the past 5 years: Almirall, Anaxys, AstraZeneca, Boehringer Ingelheim, British Lung Foundation, Chiesi, Circassia (formerly Aerocrine), Harvey Walsh, Mapi, Morningside Healthcare, Mundipharma, Mylan (formerly Meda), Napp, Novartis, Orion, Plymouth University, Regeneron, Respiratory Effectiveness Group, Roche, Sanofi, Takeda, Teva, University of East Anglia, Zentiva (a Sanofi company). DP has board membership with Amgen, AstraZeneca, Boehringer Ingelheim, Chiesi, Circassia, Mylan, Mundipharma, Napp, Novartis, Regeneron Pharmaceuticals, Sanofi Genzyme, Teva Pharmaceuticals; consultancy agreements with Amgen, AstraZeneca, Boehringer Ingelheim, Chiesi, GlaxoSmithKline, Mylan, Mundipharma, Napp, Novartis, Pfizer, Teva Pharmaceuticals, Theravance; grants and unrestricted funding for investigator-initiated studies (conducted through Observational and Pragmatic Research Institute Pte Ltd) from AKL Research and Development Ltd, AstraZeneca, Boehringer Ingelheim, British Lung Foundation, Chiesi, Circassia, Mylan, Mundipharma, Napp, Novartis, Pfizer, Regeneron Pharmaceuticals, Respiratory Effectiveness Group, Sanofi Genzyme, Teva Pharmaceuticals, Theravance, UK National Health Service, Zentiva (Sanofi Generics); payment for lectures/speaking engagements from AstraZeneca, Boehringer Ingelheim, Chiesi, Cipla, GlaxoSmithKline, Kyorin, Mylan, Merck, Mundipharma, Novartis, Pfizer, Regeneron Pharmaceuticals, Sanofi Genzyme, Teva Pharmaceuticals; payment for manuscript preparation from Mundipharma, Teva Pharmaceuticals; payment for the development of educational materials from Mundipharma, Novartis; payment for travel/accommodation/meeting expenses from AstraZeneca, Boehringer Ingelheim, Circassia, Mundipharma, Napp, Novartis, Teva Pharmaceuticals; funding for patient enrolment or completion of research from Chiesi, Novartis, Teva Pharmaceuticals, Zentiva (Sanofi Generics); stock/stock options from AKL Research and Development Ltd which produces phytopharmaceuticals; owns 74% of the social enterprise Optimum Patient Care Ltd (Australia and UK) and 74% of Observational and Pragmatic Research Institute Pte Ltd (Singapore); and is peer reviewer for grant committees of the Efficacy and Mechanism Evaluation programme, and Health Technology Assessment.
